# Effects of Dietary Fiber Intake and the Prevalence of Constipation Among Patients With End-Stage Renal Disease (ESRD) in Jeddah, Saudi Arabia: A Cross-Sectional Observational Study

**DOI:** 10.7759/cureus.62289

**Published:** 2024-06-13

**Authors:** Suhair A Abdalla, Najlaa M Al-Mana, Shahad M Hasosah, Nesreen M Alghamdi, Anwar A Alkhamesi

**Affiliations:** 1 Department of Clinical Nutrition, College of Applied Medical Sciences, King Saud bin Abdulaziz University for Health Sciences, Jeddah, SAU

**Keywords:** esrd, daily, between, management, study, hemodialysis, patient, intake, fiber, constipation

## Abstract

Rationale/background: Hemodialysis (HD) patients with end-stage renal disease (ESRD) are particularly prone to constipation, which has become a growing public health issue. Nutritional therapy, such as fiber intake, significantly influences the management of constipation. In Saudi Arabia, there is limited data on fiber consumption and its correlation with constipation management in HD patients.

Aims: The study aimed to investigate the correlation between dietary fiber intake and its effect on the prevalence of constipation in HD patients.

Materials and methods: This cross-sectional observational study of 77 ESRD patients on HD aged 18+ was conducted in a single dialysis center over six months. A questionnaire was employed to diagnose constipation (as defined by the Rome IV criteria of constipation), and seven-day food records were used to evaluate dietary fiber intake.

Results: A study found a high prevalence of constipation (53%) among participants, with a lower daily fiber intake than recommended. However, a significant relationship was found between fiber intake and constipation (p < 0.05) with lower fiber intake in constipated patients compared to non-constipated (p = 0.001).

Conclusion: The study highlights a significant link between fiber intake and constipation in HD patients, suggesting adequate daily intake of fiber was effective in preventing constipation and that nutritional counseling should include adequate daily fiber intake in medical therapy management.

## Introduction

Chronic kidney disease (CKD) is a rapidly developing global public health issue [1]. It is part of a new wave of chronic diseases that have supplanted malnutrition and infection as the primary determinants of mortality in the twentieth century [2]. In 1990, CKD was the 36th cause of death which was increased to 19th in 2013 [3]. If not managed properly, CKD may lead to end-stage renal disease (ESRD) and other cardiovascular diseases. Patients with ESRD may require replacement therapies such as peritoneal dialysis, hemodialysis (HD), or kidney transplantation which can be a considerable financial burden for both families and healthcare providers [4].

Constipation is the abnormally delayed or infrequent passage of usually dry, hardened feces. Generally described as having fewer than three bowel movements a week. It occurs when bowel movements become less frequent, and stools become difficult to pass. It happens most often due to changes in diet or routine or due to inadequate intake of fiber [5,6]. Various other factors including lack of fiber in the diet, sedentary lifestyle, certain medications, colonic motility, and several medical conditions such as hypothyroidism, diabetes, irritable bowel syndrome (IBS), and intestinal obstruction can also cause this condition [7].

Constipation has been reported to be particularly predominant in patients with CKD [8]. It’s a significant public health problem and one of the most common issues faced by patients with ESRD [9]. Constipation is not only a gastrointestinal issue, but a condition that accounts for social and economic burden [10-13]. Recent studies have revealed that the incidence of constipation among patients with CKD/ESRD, particularly those on long-term dialysis, ranged from 1.6% to 71.7% [14-18]. It has also been reported that constipation in ESRD patients is highly prevalent among patients on HD compared with those on peritoneal dialysis [19-21].

According to reports, patients with HD are more likely to suffer from constipation in Saudi Arabia. A study conducted in a single center in Saudi Arabia revealed a prevalence of 63.1% in 268 HD patients [22,23]. These complications consequently lead to many health complications and reduced quality of life [24,25]. The chronic symptoms of constipation negatively affect the quality of life of patients receiving HD treatment and impose a considerable economic and social burden. Constipation, a common health problem among HD patients, can cause a number of other complications, such as hemorrhoids and rectal prolapse if left untreated [25].

Nutritional therapy has been identified as one of the fundamental factors in managing constipation [26]. Studies have demonstrated that dietary fiber can reduce gastrointestinal symptoms associated with constipation and inflammations in healthy individuals [27]. Higher dietary fiber intake was also associated with decreased mortality in patients with CKD but not in the non-CKD population [27]. Several studies suggest the effectiveness of dietary fiber as it plays a crucial role in the treatment and control of hard stools in patients with constipation [5,6,26].

In Saudi Arabia, very limited data is available on fiber intake and its association with constipation among patients receiving HD. This is the first study conducted to assess fiber intake and its association with constipation among HD patients in a single HD center. This study aimed to determine whether fiber intake has a role in constipation among HD patients. In particular, to know the amount of fiber consumed during the day by the patient and compare it with the recommended daily allowance of fiber for this category, and to study whether the daily amount of fiber consumed by the patient, whether low, high, or sufficient, has a role in causing ESRD patients on HD to suffer from constipation. Our study will help these patients get enough fiber to manage and reduce the prevalence of constipation they have and improve their nutrition and overall health.

## Materials and methods

Patients and study design

This cross-sectional observational study was conducted in a single HD facility at the National Guard Hospital, Jeddah, Saudi Arabia. The overall study period was six months, from June 2020 to December 2020. The study population consisted of everyone who met the inclusion criteria - being over 18 years old, both sexes (males and females), having an established ESRD, getting HD, free from any other medical condition besides ESRD, not on constipation medication and being willing to sign a permission form - were included in the study population. The study did not include patients who were younger than 18 years old, were not receiving maintenance hemodialysis (MHD), had any other medical condition besides ESRD, were on constipation medication, or declined to sign a consent form. The total number of patients dialyzing at the facility was 98. Among 98 patients, 77 patients fulfilled the requirements and were included in the study, while the remaining 21 patients were excluded from the study after applying inclusion criteria. The objective of the study was revealed to the included patients before obtaining their signatures on the consent form. Nonprobability sampling (the convenient method) was used as the sampling technique, and participants were enrolled in accordance with their accessibility to and availability to meet the study's inclusion and exclusion criteria.

Method of data collection

The study's data collection was done as follows:

Firstly, information was gathered on the subject’s demographics. A validated questionnaire was created by the authors, pre-tested by a similar population (ESRD patients on HD who fulfill all the study’s criteria), and finalized to be used for this study's data collection. It was used to collect personal data including age, gender, education level, and family income.

Secondly, the questionnaire was also used to determine the constipation status based on the Rome IV criteria (The Rome IV criteria define functional constipation as fulfilling at least two of the six criteria listed: fewer than three bowel movements per week, straining during >25% of the time, lumpy or hard stools >25% of the time, sensation of anorectal obstruction >25% of the time, sensation of incomplete evacuation >25% of the time, manual maneuvers required to aid defecation >25% of the time) [28].

Thirdly, evaluated the participant’s fiber daily intake. The seven-day food records form was used to evaluate the study group's dietary intake to determine their consumption of fiber. Following this, the mean dietary intakes for seven days (which included weekends and days with no treatment or dialysis) were estimated.

The principal investigator gave specific instructions to the patients and their families on how to record their total dietary intake in a diary, including a description of the foods and amounts ingested (in household measurements), comprising both the food items and serving sizes (using seven-day food record forms for dialysis food questionnaires). At the end of the recording period, the principal investigator reviewed all food records to look for any missing data and clarify data entries. The MyPyramid Tracker program online (United States Department of Agriculture (USDA), Alexandria, USA) and electronic food composition tables (for traditional foods) were used to estimate the amount of fiber consumed. Food items in household measures were then translated to weights. For all subjects, the daily fiber consumption was taken to be the average for the seven days.

Finally, the intake of participants was compared with the recommended daily fiber intake from RDIs (recommended dietary intake forms), which is the amount of fiber that is recommended by the National Kidney Foundation for ESRD patients on HD.

Statistical analysis

The statistical software program JMP (John’s Macintosh Project; SAS Institute Inc., Cary, USA) was used to conduct all statistical analyses. For categorical variables, the results are shown as frequencies and percentages, and for all continuous variables, the mean (±) standard deviation (SD) was determined. Data were evaluated for comparison and differences between means using the ‘t’ test and the chi-square test (χ^2^). It was deemed significant if p < 0.05.

## Results

Demographics of the participants

Table 1 displays the demographic details of the study participants. The analysis revealed that the proportion of men (56%) was higher as compared to women (44%) in our study. Family income of most of the participants ranged from 2000 Saudi Riyal (SAR) to less than SAR 5000 (38%), followed by less than SAR 2000 (36%), 16% of participants had above SAR 10000, and 10% from SAR 5000 to 10000. Most participants were retired (40%), followed by housewives (30%), and the rest were unemployed (17%). The results about the participant’s educational backgrounds showed that 48% of participants were illiterate, followed by primary education (25%), and the rest were intermediate and postgraduate. According to the participants' age distribution, most of the patients (53%) were over the age of 65. Only 3% of respondents were discovered to be between the ages of 18 and less than 30. The next group was (27%) in the age range of 46 to 65. The median age of the participants was 63 years.

**Table 1:** The Demographic Characteristics of the Study Participants (n=77) Data are represented as n(%); *Saudi Riyals

Parameter	Frequency	Percentage	P-value
Sex			
Female	34	44%	0.44156
Male	43	56%	0.55844
Family income/SAR*			
2000-less than 5000	29	38%	0.37662
5000-10000	8	10%	0.1039
Above 10000	12	16%	0.15584
Less than 2000	28	36%	0.36364
Occupation			
Businessman	1	1%	0.01299
Employee	9	12%	0.11688
Housewife	23	30%	0.2987
Retired	31	40%	0.4026
Unemployed	13	17%	0.16883
Education			
Illiterate	37	48%	0.48052
Intermediate	7	9%	0.09091
Postgraduate	1	1%	0.01299
Primary	19	25%	0.24675
Secondary	7	9%	0.09091
University	6	8%	0.07792
Age			
18-less than 30	2	3%	0.02597
30-45	13	17%	0.16883
46-65	21	27%	0.27273
Above 65	41	53%	0.53247

Prevalence of constipation

**Figure 1:**
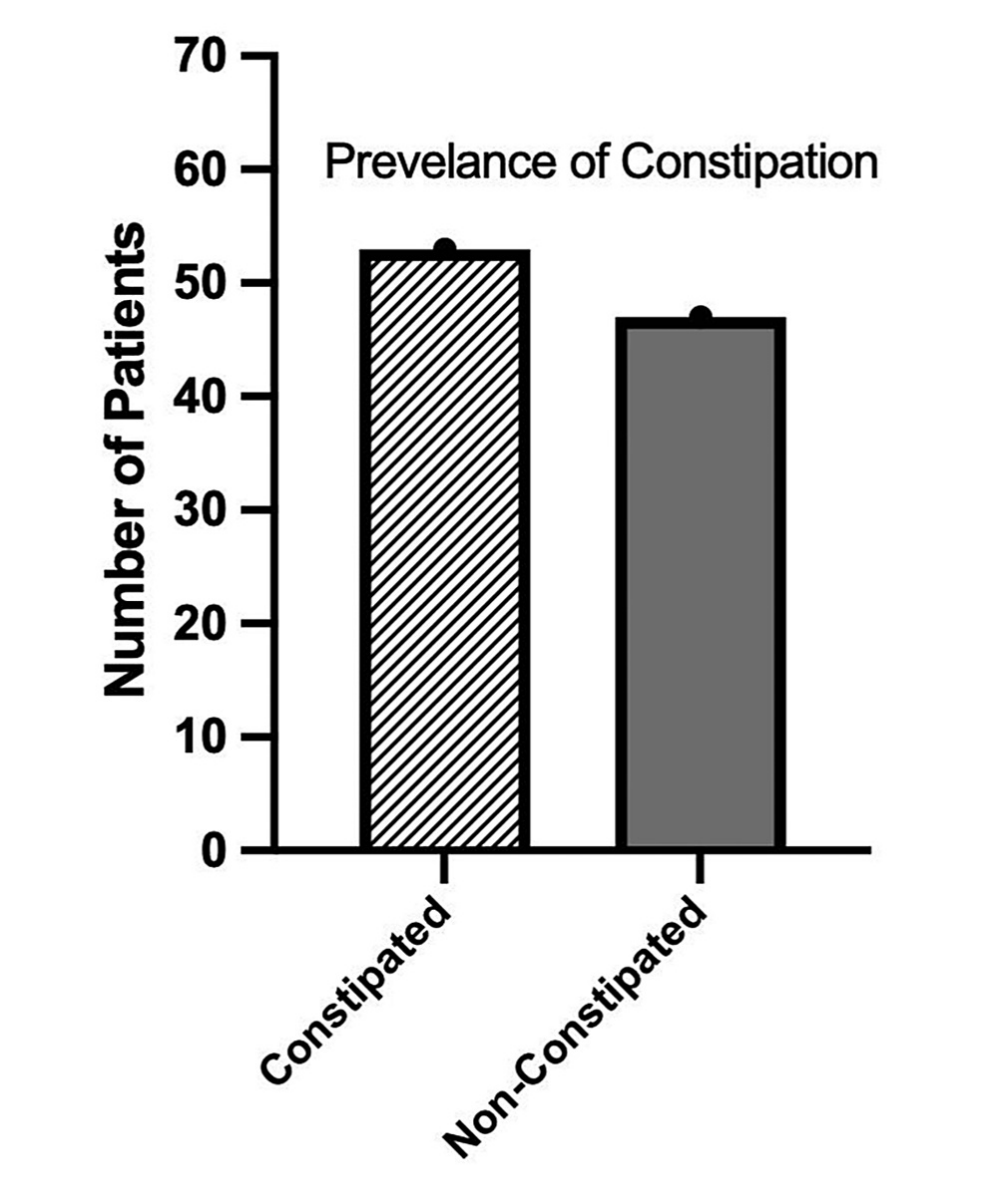
Graphical Representation of the Prevalence of Constipation

Figure 1 explains the prevalence of constipation among the participants during the study. The study shows that 41 (53%) of the participants had constipation, while only 36 (47%) were not constipated (p = 0.53247 and 0.46753, respectively).

Fiber intake

**Figure 2:**
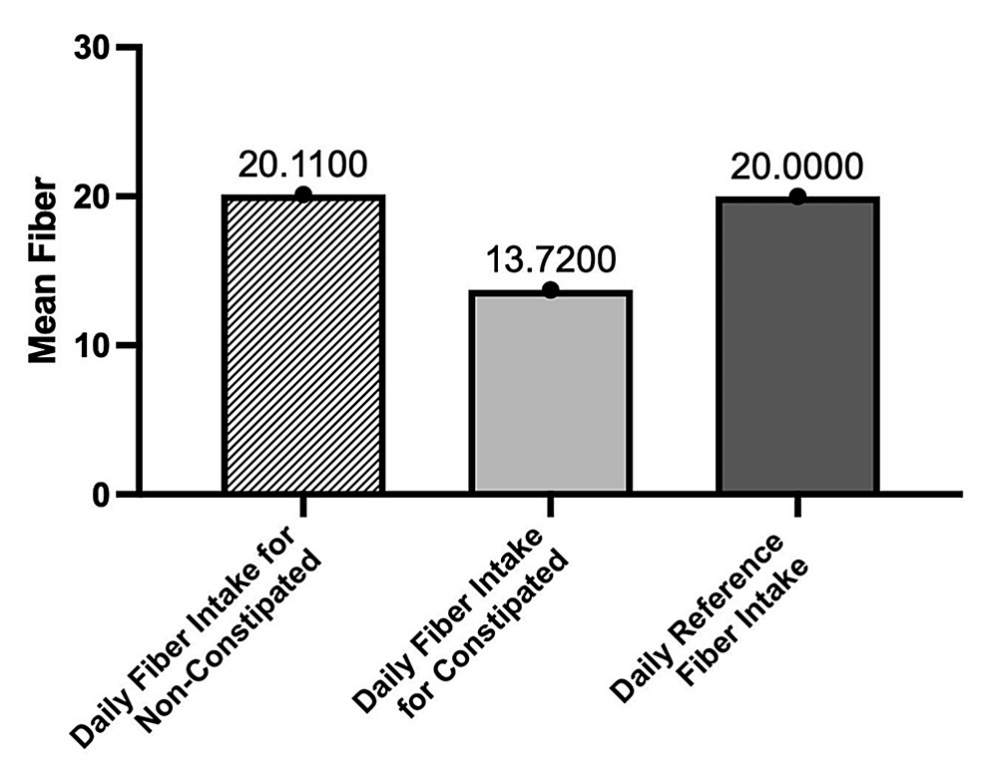
The Comparison Between Daily Fiber Reference Intake (g/day) and Daily Fiber Intake (g/day) During the Study Results are expressed as mean ± standard deviation, p < 0.05.

Figure 2 shows the daily fiber intake (g/day) for the whole group of participants during the study. The constipated patients showed a mean daily fiber intake (g/day) of 13.72 (±6.64), while the mean intake of non-constipated patients was 20.11 (±8.96) (p = 0.0009 and 0.0024 respectivly). The mean fiber intake was highly significant in both constipated and non-constipated patients (p < 0.05). There was a significant relationship between fiber intake and constipation. As shown by the study (p < 0.05), the mean daily fiber reference intake (g/day) is 20 g for both constipated and non-constipated patients. However, the results show that the mean daily fiber intake (g/day) was 13.72g for constipated patients, which was significantly lower as compared to 20.11g for non-constipated patients in the study (p = 0.001 in both). This necessitates the urgent need to improve the fiber intake in patients with constipation.

## Discussion

The study looked at 77 ESRD patients on MHD attending the renal dialysis unit in a single hospital. The goal of the study was to evaluate the association between plant-based fiber intake and the prevalence of constipation among HD patients. The study was also employed to assess the prevalence of constipation among HD patients. Our results from this study demonstrated a high prevalence of constipation (53%). Similar to our findings in this study, other studies have shown that the prevalence of patients with HD is approximately 53%, ranging from 8% to 57% [20]. A study that included 268 HD patients reported 63.1% constipation which is in line with the results of our study [22].

The presence of constipation among HD patients negatively affects their quality of life and has been associated with many health complications. International guidelines suggest that all patients with MHD must receive a recommended daily dietary fiber [5] of 20 to 30 g per day to decrease the incidence of constipation [6]. Our results revealed a low fiber intake (16.71 (±8.39) g/day) for the whole group of participants in the study. However, constipated patients showed a lower mean daily fiber intake of 13.72 (±6.64) g/day compared with the mean intake of fiber for non-constipated patients, which was 20.11 (±8.96) g/day.

The comparison between the mean daily fiber reference intake of 20 g/day and the mean daily fiber intake (g/day) of the participants during the study was significantly different. Our study found less daily fiber intake (13.72 (±6.64) g/day) among participants with constipation (p = 0.0009). The non-constipated patients were found to be taking daily fiber intake within the normal fiber reference intake value (20.11 (±8.96) g/day), as recommended by the international guidelines (p = 0.0024). Our study approves the strong relationship between fiber intake and constipation (p < 0.05). Other studies have demonstrated similar results indicating that the intake of 23g of added fiber per day resulted in an increased stool frequency in HD patients [19].

The low fiber intake below the recommended daily reference intake among patients with MHD is a leading cause of constipation [6,26]. It influences the quality of life and imposes significant economic and social burdens [18]. Our findings are in agreement with other studies that reported lower mortality risk in CKD patients who were taking a plant-based, fiber-rich diet [29]. Our results also demonstrated that fiber intake improved the management of constipation among HD patients which is in line with the results of a meta-analysis that concluded the improvement of constipation and bowel movement when fiber was taken by the patients [29,30].

A limited number of studies have evaluated the association between fiber intake and the prevalence of constipation among HD patients in the world, especially in Saudi Arabia. In Southern Brazil, a multi-center cross-sectional study was conducted among HD patients (n = 305) which observed an inverse association between constipation and usual intake of fiber [30]. Another six-week, single-blind study using isomalto-oligosaccharide in the treatment of constipation in HD patients reported that consuming foods with added fiber may be an effective means of increasing fiber intake and improving stool frequency in individuals with CKD [30].

Several other studies have also shown that most HD patients who suffer from constipation can be managed by the ingestion of fiber in different ways and different dietary regimens [26,27]. Our study demonstrated the same results of a strong relationship between constipation and lack of fiber intake.

Overall, our study showed that proper fiber intake in the form of the daily recommended reference intake will help to prevent constipation in HD patients, and a lower fiber intake of less than the daily reference intake will lead to constipation among patients on MHD. It is therefore strongly suggested that all patients with moderate-to-severe MHD should consume a sufficient amount of dietary fiber in accordance with the recommended daily intake in order to maintain and reduce constipation and associated complications.

Our study has a very small p-value that indicates a strong relationship between developing constipation and increased fiber intake. Moreover, a three-day food record was collected in a consistent manner which increased the accuracy and the validity of the collected data. In addition, the response rate was high, and the majority of the participants were responsive and collaborative which increased the quality of data. However, we are concerned about the response bias which can have a large impact on the validity of the questionnaire. Also, the study is an observational cross-sectional study which is considered one of the weak types of studies. Additionally, a few participants who responded weakly tended to record less and increase the time duration. Likewise, food record analysis was time-consuming. Last, in the present study we have a small sample size and, therefore, may lack generalization to the population. Based on all findings, our study showed that proper fiber intake in the form of daily recommended reference intake will help to prevent constipation in HD patients, and on the other hand, the lower fiber intake of less than the daily reference intake will lead to constipation among patients on MHD.

## Conclusions

Our study demonstrated that there is a significant relationship between fiber intake and constipation among HD patients. The daily fiber intake below the reference recommended value for HD patients is a leading cause of constipation. An adequate intake of fiber per day was effective in preventing constipation among patients. Therefore, nutritional counseling by qualified dietitians that helps in designing an individual dietary plan with adequate fiber intake should be mandatory in renal units. Future studies are required to fill in the knowledge gaps and offer solutions for patients with CKD through a high-fiber diet.

## References

[1] Cano AE, Neil AK, Kang JY (2007). Gastrointestinal symptoms in patients with end-stage renal disease undergoing treatment by hemodialysis or peritoneal dialysis. Am J Gastroenterol.

[2] Zoccali C, Kramer A, Jager KJ (2010). Epidemiology of CKD in Europe: an uncertain scenario. Nephrol Dial Transplant.

[3] GBD 2013 Mortality and Causes of Death Collaborators (2015). Global, regional, and national age-sex specific all-cause and cause-specific mortality for 240 causes of death, 1990-2013: a systematic analysis for the Global Burden of Disease Study 2013. Lancet.

[4] Ikee R, Sasaki N, Yasuda T, Fukazawa S (2020). Chronic kidney disease, gut dysbiosis, and constipation: a burdensome triplet. Microorganisms.

[5] Raymond JL, Morrow K (2022). Medical therapy for renal disorders. Krause and Mahan’s Food and the Nutrition Care Process, 16th Edition.

[6] Byham-Gray L, Stover J, Wiesen K (2013). A Clinical Guide to Nutrition Care in Kidney Disease (Second Edition). https://ocul-uwo.primo.exlibrisgroup.com/discovery/fulldisplay?context=L&vid=01OCUL_UWO:UWO_DEFAULT&search_scope=MyInst_and_CI&tab=Everything&docid=alma991009015309705163.

[7] Forootan M, Bagheri N, Darvishi M (2018). Chronic constipation: a review of literature. Medicine (Baltimore).

[8] Lu CY, Chen YC, Lu YW, Muo CH, Chang RE (2019). Association of constipation with risk of end-stage renal disease in patients with chronic kidney disease. BMC Nephrol.

[9] Ikee R, Yano K, Tsuru T (2019). Constipation in chronic kidney disease: it is time to reconsider. Ren Replace Ther.

[10] Bharucha AE, Pemberton JH, Locke GR 3rd (2013). American Gastroenterological Association technical review on constipation. Gastroenterology.

[11] Sumida K, Molnar MZ, Potukuchi PK (2019). Constipation and risk of death and cardiovascular events. Atherosclerosis.

[12] Sumida K, Yamagata K, Kovesdy CP (2020). Constipation in CKD. Kidney Int Rep.

[13] Zuvela J, Trimingham C, Le Leu R, Faull R, Clayton P, Jesudason S, Meade A (2018). Gastrointestinal symptoms in patients receiving dialysis: a systematic review. Nephrology (Carlton).

[14] Zhang J, Huang C, Li Y (2013). Health-related quality of life in dialysis patients with constipation: a cross-sectional study. Patient Prefer Adherence.

[15] Chong VH, Tan J (2013). Prevalence of gastrointestinal and psychosomatic symptoms among Asian patients undergoing regular hemodialysis. Nephrology (Carlton).

[16] Ikee R, Toyoyama T, Endo T, Tsunoda M, Hashimoto N (2016). Clinical factors associated with constipation in hemodialysis patients. Int Urol Nephrol.

[17] Mitrovic M, Majster Z, Damjanović T (2015). The prevalence, severity and diversity of gastrointestinal symptoms in hemodialysis and peritoneal dialysis patients. Nephrol Dial Transplant.

[18] Lee A, Lambert K, Byrne P, Lonergan M (2016). Prevalence of constipation in patients with advanced kidney disease. J Ren Care.

[19] Dong R, Guo ZY, Ding JR, Zhou YY, Wu H (2014). Gastrointestinal symptoms: a comparison between patients undergoing peritoneal dialysis and hemodialysis. World J Gastroenterol.

[20] Zhang L, Tang F, Wang F (2022). The prevalence of constipation in end-stage kidney disease patients: a cross-sectional observation study. Medicine (Baltimore).

[21] Ramos CI, Nerbass FB, Cuppari L (2022). Constipation in chronic kidney disease: it is time to bridge the gap. Kidney Dial.

[22] Yasuda G, Shibata K, Takizawa T, Ikeda Y, Tokita Y, Umemura S, Tochikubo O (2002). Prevalence of constipation in continuous ambulatory peritoneal dialysis patients and comparison with hemodialysis patients. Am J Kidney Dis.

[23] Masakane I, Taniguchi M, Nakai S (2018). Annual dialysis data report 2016, JSDT Renal Data Registry. Ren Replace Ther.

[24] Murtagh FE, Addington-Hall J, Higginson IJ (2007). The prevalence of symptoms in end-stage renal disease: a systematic review. Adv Chronic Kidney Dis.

[25] Width M, Reinhard T (2020). Chapter 12. Chronic kidney disease. The Essential Pocket Guide for Clinical Nutrition.

[26] Krishnamurthy VM, Wei G, Baird BC (2012). High dietary fiber intake is associated with decreased inflammation and all-cause mortality in patients with chronic kidney disease. Kidney Int.

[27] Salmean YA, Zello GA, Dahl WJ (2013). Foods with added fiber improve stool frequency in individuals with chronic kidney disease with no impact on appetite or overall quality of life. BMC Res Notes.

[28] Kelly JT, Palmer SC, Wai SN, Ruospo M, Carrero JJ, Campbell KL, Strippoli GF (2017). Healthy dietary patterns, and risk of mortality and ESRD in CKD: a meta-analysis of cohort studies. Clin J Am Soc Nephrol.

[29] Sumida K, Molnar MZ, Potukuchi PK (2017). Constipation and incident CKD. J Am Soc Nephrol.

[30] Jani B, Marsicano E (2018). Constipation: evaluation and management. Mo Med.

